# Allometric wing growth links parental care to pterosaur giantism

**DOI:** 10.1098/rspb.2023.1102

**Published:** 2023-07-26

**Authors:** Zixiao Yang, Baoyu Jiang, Michael J. Benton, Xing Xu, Maria E. McNamara, David W. E. Hone

**Affiliations:** ^1^ School of Biological, Earth and Environmental Sciences, University College Cork, Cork T23 TK30, Ireland; ^2^ Environmental Research Institute, Ellen Hutchins Building, Lee Road, Cork T23 XE10, Ireland; ^3^ Center for Research and Education on Biological Evolution and Environments, School of Earth Sciences and Engineering, Nanjing University, Nanjing 210023, People's Republic of China; ^4^ School of Earth Sciences, University of Bristol, Life Sciences Building, Tyndall Avenue, Bristol BS8 1TQ, UK; ^5^ Center for Vertebrate Evolutionary Biology, Yunnan University, Kunming 650031, People's Republic of China; ^6^ Key Laboratory of Vertebrate Evolution and Human Origins, Institute of Vertebrate Paleontology and Paleoanthropology, Chinese Academy of Sciences, Beijing 100044, People's Republic of China; ^7^ School of Biological and Behavioural Sciences, Queen Mary University of London, Mile End Road, London E1 4NS, UK

**Keywords:** giantism, growth, allometry, flight, Pterosauria, wing shape

## Abstract

Pterosaurs evolved a broad range of body sizes, from small-bodied early forms with wingspans of mostly 1–2 m to the last-surviving giants with sizes of small airplanes. Since all pterosaurs began life as small hatchlings, giant forms must have attained large adult sizes through new growth strategies, which remain largely unknown. Here we assess wing ontogeny and performance in the giant *Pteranodon* and the smaller-bodied anurognathids *Rhamphorhynchus*, *Pterodactylus* and *Sinopterus*. We show that most smaller-bodied pterosaurs shared negative allometry or isometry in the proximal elements of the fore- and hindlimbs, which were critical elements for powering both flight and terrestrial locomotion, whereas these show positive allometry in *Pteranodon*. Such divergent growth allometry typically signals different strategies in the precocial–altricial spectrum, suggesting more altricial development in *Pteranodon*. Using a biophysical model of powered and gliding flight, we test and reject the hypothesis that an aerodynamically superior wing planform could have enabled *Pteranodon* to attain its larger body size. We therefore propose that a shift from a plesiomorphic precocial state towards a derived state of enhanced parental care may have relaxed the constraints of small body sizes and allowed the evolution of derived flight anatomies critical for the flying giants.

## 1. Introduction

The pterosaurs are a diverse clade of Mesozoic flying reptiles that achieved the largest body sizes for volant animals in Earth's history. Pterosaur body size evolution has been viewed as a two-stage process: early pterosaurs in the Triassic and Jurassic were relatively small animals, most with 1–2 m wingspans, while the later pterodactyloids experienced a sustained, multi-lineage body size increase through the Cretaceous, giving rise to flying giants with 7–10 m wingspans by the end of the Cretaceous [1–3]. A few specimens of large pterosaurs are, however, known from the Jurassic (e.g. [4–6]), suggesting that larger sizes appeared earlier than previously recognized [6].

Regardless of adult size, all pterosaurs began life as small hatchlings just a few tens of centimetres in wingspan [1,7]. This is due to the physical constraints on pterosaur egg size, which were imposed by the weakly or non-calcified soft shell and the size of the pelvic opening [1,7]. Giant pterosaurs therefore must have used different postnatal developmental strategies from small-bodied taxa to allow larger adult sizes, i.e. faster growth and/or a longer growth period.

It has been hypothesized that modifications to the body plan, particularly the derived anatomies seen in pterodactyloids, may have allowed improved flight performance, and thereby enabled larger body sizes [8–10]. Specifically, giant pterosaurs may have been more efficient during powered flight and/or (unpowered) gliding, which is commonly used by large birds today [11]. Venditti *et al*. [12] recently demonstrated 150 Myr of progressive enhancement of both powered and gliding flight performance in pterosaurs, evidenced by sustained increase in flight efficiency and decrease in sinking rate (the speed of altitude loss during gliding), respectively; pterosaur body size [2] did not, however, increase in tandem with flight efficiency, suggesting additional factors driving body size evolution. Moreover, while the adults of the late-diverging, giant forms had better flight performance compared with earlier, smaller pterosaurs [12], such differences may have been dynamic during growth. This is because wing planforms could have changed considerably during allometric growth (where body proportions change owing to growth rate differentials among different body parts through ontogeny). Critically, it remains unclear whether juveniles of the giant pterosaurs had a more aerodynamic wing planform than similarly sized smaller taxa; this would have enabled such juveniles to extend their growth and attain a larger adult size.

Indeed, allometric growth in various limb elements has been shown in diverse pterosaurs. Examples include (i) negative allometric growth of the proximal portion of the forelimb and positive allometric growth of the metacarpal IV in *Pterodaustro* [13]; (ii) negative allometry in the tibia relative to the femur and positive allometry in metacarpal IV relative to the radius/ulna and to the first wing phalanx (WP1) in *Pteranodon* [14]; (iii) positive allometry in metacarpal IV and proximal wing phalanges relative to the antebrachium and to distal wing phalanges in *Pterodactylus* [15]; (iv) negative allometry in the humerus, radius and metacarpal IV and positive allometry in WP2–3 relative to body length in *Rhamphorhynchus* [16]; and (v) positive allometric growth in distal wing phalanges of anurognathids [17]. Such allometric growth would have caused various degrees of wing planform changes during growth, which may have had a more profound impact on giant pterosaurs given the magnitude of their size change during ontogeny.

Allometric growth patterns could also potentially inform on the precocial–altricial developmental spectrum. In extant birds and mammals, parental care enables altricial species to have higher growth rates than their precocial counterparts [18–20]. Locomotor agents (i.e. legs and wings) of altricial species are generally small and have low tissue and functional maturity at hatching (or birth); the locomotor elements then grow faster than body size increase, illustrating positive allometric growth, and eventually function at proportions similar to the adult [21–23]. By contrast, precocial species have well-developed, functional locomotors at or soon after hatching (or birth); their locomotors often either grow in tandem with body size (i.e. isometric growth) to permit function at early developmental stages, or become relatively smaller during growth (i.e. negative allometry), often associated with an ontogenetic change in locomotion [21–24].

Critically, precocial development can constrain adult size by inducing early termination of growth and ontogenetic canalization, a process that retains juvenile phenotypes into adulthood [21,23]. Although (super-)precocial flight has been suggested for many pterosaurs based on, in part, (near-)isometric wing growth [1,7,8,16,25], it remains unknown whether this developmental strategy applies to any giant pterosaur.

Here we use a multivariate approach to assess changes in wing planform and flight performance during growth for five pterosaurs from diverse lineages and representing disparate adult sizes. We reconstruct wing shape and area during growth from early juveniles (with 0.3 m wingspan) to giant adults (7 m wingspan, which is hypothetical for the small-bodied taxa) and apply a biophysical model of powered and gliding flight to these wing traits to determine variation in flight performance during growth. We use these data to test two hypotheses: (i) juveniles of giant pterosaurs had an aerodynamically superior wing planform, ultimately allowing further growth than pterosaurs with smaller adult sizes; (ii) giant pterosaurs were altricial, thereby exceeding the body size constraints on their precocial counterparts.

## 2. Material and methods

### (a) Allometric analysis

Measurements of 13 key skeletal dimensions were collected for allometric analysis: 1, skull length; 2, neck length; 3, tail length; 4, humerus length; 5, ulna or radius length; 6, wing metacarpal length; 7–10, lengths of WP1–4, respectively; 11, femur length; 12, tibia length; 13, wingspan (= combined length of humerus + ulna/radius + metacarpal IV + WP1–4) × 2.1) [26]. Measurements of *Rhamphorhynchus muensteri* (*sensu* Bennett [27]), *Pterodactylus antiquus* (*sensu* Bennett [15] and Vidovic & Martill [28]), *Sinopterus dongi* (*sensu* Pêgas *et al*. [29]) and *Pteranodon* (*sensu* Bennett [14]) were taken from Hone *et al*. [16] (88 specimens), Wellnhofer [30] (22 specimens), Pêgas *et al*. [29] (and references therein; 10 specimens) and Bennett [14,31] (59 specimens), respectively (see electronic supplementary material, data S1 for detailed measurements for each specimen). Measurements of *Pteranodon* include two species, *Pteranodon sternbergi* and *Pteranodon longiceps*, because they do not differ in their postcranial skeletal anatomy [14] and only postcranial measurements are included in this analysis.

Among the specimens with complete preservation of forelimb elements, the wingspan ranges are 0.30–1.28 m for *Rhamphorhynchus*, 0.19–0.74 m for *Pterodactylus*, 0.81–2.17 m for *Sinopterus* and 3.91–6.37 m for *Pteranodon*. Incomplete preservation of forelimb elements prevents calculation of wingspan. Based on the length of individual elements, however, the wingspan of some incomplete specimens is likely to lie beyond the stated range for each taxon above. This likely difference in wingspan between complete and incomplete specimens is particularly pronounced for *Pteranodon*, for which most specimens are incomplete. At least five incomplete *Pteranodon* specimens probably fall below the wingspan range (i.e. less than 3.91 m wingspan) and the smallest (and incomplete) specimen had an estimated wingspan of 1.76 m [31]. Although smaller specimens of *Pteranodon* (i.e. less than 1.76 m in wingspan) are currently unknown, the available specimens cover a broad range of sizes, allowing investigation of growth allometry.

Measurements for each taxon were subjected to allometric analysis using a multivariate approach of principal components analysis following Yang *et al*. [17] (see electronic supplementary material for details); skeletal dimensions with no available data were not included in the analysis. Growth allometry of anurognathids was taken from Yang *et al*. [17] for comparison and for subsequent ontogenetic reconstruction of wing planforms.

### (b) Ontogenetic reconstruction of wing planforms

Results on growth allometry enabled us to extrapolate skeletal dimensions at various wingspans in growth and at hypothetical larger sizes (i.e. beyond actual adult sizes), by using the following equation:

$$Y_{2}=Y_{1}{\left(\frac{Z2}{Z1}\right)}^{\frac{b_{y}}{b_{z}}}.$$

**Figure 1.**
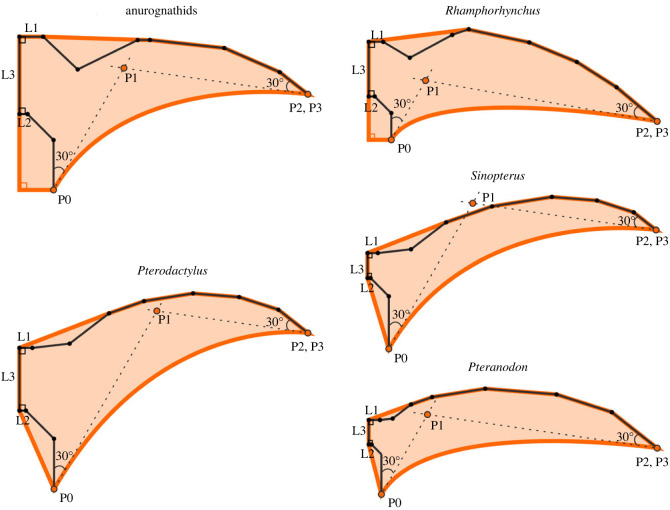
Wing planforms (orange outlines) modelled for the studied pterosaurs using taxon-specific postures. The black dots and solid lines denote the studied skeletal dimensions; the orange dots and dashed lines define the trailing edges (see Material and methods text for details). Taxa are not to scale.

*Y*_1_ and *Y*_2_ are dimensions of a skeletal element (e.g. humerus length, ulna length, etc.) at wingspans *Z*_1_ and *Z*_2_, respectively; *b_y_* and *b*_z_ are allometric coefficients of the skeletal dimension and wingspan, respectively. Specimen-based measurements were used for *Y*_1_ and *Z*_1_, based on which *Y*_2_ was extrapolated at wingspans from 0.3 to 7 m. The specimens used were SMNS 81928 [32], Wellnhofer 1975 #20, Wellnhofer 1970 #1, IVPP V 13363 and AMNH 6158 (electronic supplementary material, data S1) for anurognathids, *Rhamphorhynchus*, *Pterodactylus*, *Sinopterus* and *Pteranodon*, respectively. In order to define trunk sizes, we extrapolated the distance between humeral head and body midline (L1), the distance between femoral head and body midline (L2) and the distance between forelimb and hindlimb attachments along the midline (L3) (figure 1); these three dimensions were calculated by assuming isometric growth. In addition, WP3–4 were treated as a single dimension (i.e. using the combined length and the average posture of the two bones) for anurognathids following Yang *et al*. [17], because the presence of WP4 in juvenile *Anurognathus* remains controversial [33].

Forelimb postures of anurognathids and *Rhamphorhynchus* were modelled using the wing reconstructions in [34]. For *Pteranodon*, we used the forelimb posture in [35] as the anterior sweep position is more aerodynamic whereby the centre of mass and centre of pressure coincide. To date, reconstructions of the wing of *Sinopterus* do not consider aerodynamics; we modelled its posture based on the tapejarid *Tupuxuara* [34]. Because *Tupuxuara* and *Pterodactylus* have similar positions of centre of mass and wing centroid to those in *Pteranodon* [34], we applied the *Pteranodon* forelimb posture to both *Sinopterus* and *Pterodactylus*.

Two extreme positions are proposed for hindlimbs in previous pterosaur reconstructions: (i) hindlimbs extended directly posteriorly (‘straight-legged’ model) and (ii) femur extended laterally and tibia posteriorly (‘bat-like’ model) [36]. Padian *et al*. [36] argued that neither model is realistic owing to the range of motion at hindlimb joints and thus intermediate hindlimb positions (as used in most recent studies) are more likely. Here we oriented the femur at 45° to the acetabulum and the tibia, parallel to the body midline (figure 1).

We used an ankle attachment for the trailing edge of the main wing membrane (i.e. the brachiopatagium; as in [37]). Since the curvature of the trailing edge remains unclear, the trailing edge was defined using the same method for all five pterosaur taxa to prevent artificially imposed variations. It was taken as a Bézier curve, which was mathematically defined by four control points (P0–P3 in figure 1). P1 and P2 define the curvature; P0 and P3 define the ends of the curve located at the distal termini of tibia and wing finger, respectively. P1 was placed at the intersection of two lines oriented at 30° to tibia and the distalmost phalanx of the wing finger, respectively; P2 was placed at the same position as P3 (figure 1). Uropatagium reconstructions for basal and pterodactyloid pterosaurs follow those of Witton [38].

Wing planform changes during growth from 0.3 to 7 m wingspan were then modelled based on the extrapolated dimensions, limb postures and wing membrane definitions. The area of the resultant wing planform was measured using the image processing freeware ImageJ (available at http://rsbweb.nih.gov/ij/); wing area was calculated as twice the measured area for a single wing. Wing aspect ratio was then calculated as wingspan^2^/wing area.

We also modelled the wing planforms using a neutral posture to test the effects of taxonomic variation in posture on our aerodynamic models. The neutral posture is the average limb posture of the five pterosaurs; each pterosaur has the same pose with the leading edge fundamentally perpendicular to the long axis of the body (electronic supplementary material, figure S2).

### (c) Flight performance calculations

Following the methods outlined in Venditti *et al*. [12], performance of powered and gliding flight at wingspans from 0.3 to 7 m was estimated for each pterosaur via an actuator-disc-based biophysical model. Unlike Venditti *et al*. [12], who used a modified version of Pennycuick's [39] flight model, we used the ‘afpt’ package [40] in R v. 4.1.2, because ‘afpt’ is readily accessible and it was also developed from, and provides more accurate estimations [40] than, Pennycuick's [39] flight model.

Flight performance was estimated using body mass, wingspan and wing area. There is a significant positive correlation between pterosaur body mass and wingspan, but previous studies using different approaches have yielded different scaling equations for body mass estimation. For comparison, here we used the equations recovered by two different approaches (by Witton [38] and Henderson [34], respectively) and generated two sets of body mass estimations for subsequent aerodynamic modelling. The equations are *M*_non-pterodactyloid pterosaurs_ = 0.681 × wingspan^2.807^ and *M*_pterodactyloids_ = 0.519 × wingspan^2.550^ [38] and *M*_non-pterodactyloid pterosaurs_ = 0.3 × wingspan^2.74^ and *M*_pterodactyloids_ = 0.315 × wingspan^2.56^ [34], where *M* is body mass (kg). Wing loading was calculated as *M*/wing area (kg m^−2^).

The model produced a U-shaped power-to-airspeed relationship from which a minimum power speed (*V*_mp_) can be calculated, that is, the least energetically expensive flight speed. The model also estimated the total aerodynamic drag (*D*), resulting from the addition of the induced, parasite and profile drags. Following Venditti *et al*. [12], two indices of flight performance were calculated. The first index is the efficiency of flight (kg m J^−1^), which is the inverse of the cost of transport (COT^−1^); the COT is the metabolic energy required to move a unit mass a unit distance at the least energetically expensive travel speed. The efficiency of flight index was calculated as (*V*_mp_ × *M*)/*P*_BMR_, where *P*_BMR_ was estimated as 3.277M^0.624^ (J s^−1^). The second index is the sinking rate *V*_z_ (m s^–1^), which is the speed of altitude loss during gliding; it was calculated as *D* × *V*_mp_/*M* × ***g***, where ***g*** is the gravitational acceleration, equal to 9.81 m s^−2^. An additional index, glide ratio, was calculated as the ratio of forward speed to sinking rate; it is a measure of the gain in forward advance given the loss in altitude [39] and therefore was used here as an indicator for gliding performance.

To test whether the ontogenies of the five pterosaur taxa are significantly different in terms of powered and gliding flight performance, a pair-wise permutation test was performed for COT^−1^ and glide ratio, respectively. We used the *symmetry_test* function in the R package ‘coin’ [41], which treats the index values, at each wingspan, and for both of the paired pterosaur taxa, as paired data. Flight performance between taxa was considered as different at the 0.05 significance level; otherwise, a comparable level of performance was assumed.

## 3. Results

### (a) Growth allometry of limb elements

**Figure 2.**
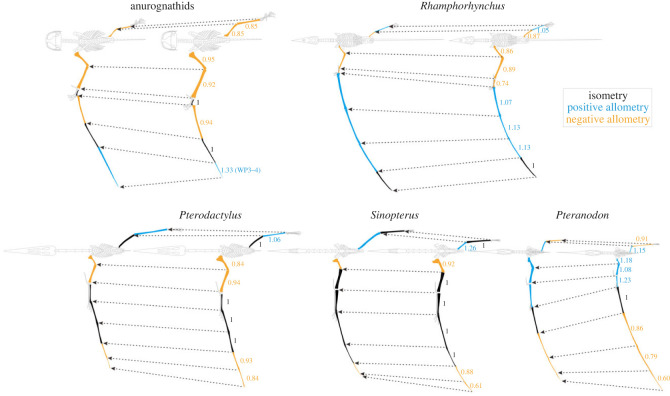
Growth allometry of limb elements in the studied pterosaurs. Allometric growth of limbs from early juveniles of 0.3 m wingspan (right) to adults of 7 m wingspan (left); note that the adult sizes are hypothetical for all pterosaurs except *Pteranodon*. Skeletal reconstructions are modified from those in Wellnhofer [30], Bennett [14,32], Hone *et al*. [16] and Beccari *et al*. [42]. Allometric coefficients are indicated for the corresponding limb elements; positive and negative allometry and isometry are indicated in blue, orange and black, respectively.

Allometric analysis reveals a unique growth pattern for each pterosaur (figure 2 and table 1; electronic supplementary material, table S1). Despite this, there are common growth trajectories shared by the smaller-bodied pterosaurs (i.e. anurognathids, *Rhamphorhynchus, Pterodactylus* and *Sinopterus*) that contrast with *Pteranodon*. Among the smaller-bodied pterosaurs, the proximal portion of the forelimb shows overall negative allometric growth, which is the combined effect of negative allometry in the humerus and ulna/radius and negative allometry or isometry in metacarpal IV; on the other hand, *Pteranodon* shows positive allometry in all of these elements. A similar contrast is also seen in the hindlimb, where the femur shows either negative allometry or isometry in most smaller-bodied pterosaurs but positive allometry in *Pteranodon*; the only exception of the smaller-bodied pterosaurs is *Sinopterus,* which also shows positive allometry in the femur.

**Table 1.** Growth allometry of limb elements. Values for anurognathids were taken from Yang *et al*. [17]. Abbreviations: A, allometry; AC, allometric coefficient; CI, one-tailed 95% confidence interval; Fe, femur; Hu, humerus; McIV, metacarpal IV; Ra, radius; Ti, tibia; Ul, ulna; WP1–4, wing phalanges 1–4; WS, wingspan. See electronic supplementary material, table S1 for growth allometry of other dimensions (skull, neck and tail length).

		Hu	Ul/Ra	McIV	WP1	WP2	WP3	WP4	WS	Fe	Ti
*Rhamphorhynchus*	AC	0.86	0.89	0.74	1.07	1.13	1.13	1.01	1.02	0.87	1.05
	CI	<0.88	<0.90	<0.78	>1.06	>1.12	>1.11	>0.97	>1.01	<0.91	>1.03
	A	−	−	−	+	+	+	=	+	−	+
*Pterodactylus*	AC	0.84	0.94	1	1	0.99	0.93	0.84	0.94	0.99	1.06
	CI	<0.87	<0.97	<1.03	<1.04	<1.03	<0.97	<0.89	<0.97	<1.02	>1.04
	A	−	−	=	=	=	−	−	−	=	+
*Sinopterus*	AC	0.92	1.04	0.97	1.10	1.03	0.88	0.61	0.95	1.26	1.04
	CI	<0.98	>0.98	<1.17	>0.98	>0.97	<0.94	<0.97	<0.97	>1.10	>0.94
	A	−	=	=	=	=	−	−	−	+	=
*Pteranodon*	AC	1.18	1.08	1.23	1.03	0.86	0.79	0.6	0.99	1.15	0.91
	CI	>1.16	>1.06	>1.14	>0.97	<0.93	<0.84	<0.74	<0.99	>1.14	<0.93
	A	+	+	+	=	−	−	−	−	+	−
anurognathids	AC	0.95	0.92	1	0.94	1	1.33		1	0.85	0.85
	A	−	−	=	−	=	+		=	−	−

The allometric growth patterns (figure 2 and table 1; electronic supplementary material, table S1) recovered by our multivariate approach are consistent with previous reports of allometry that used bivariate methods. These reports conclude that: (i) the ontogeny of *Rhamphorhynchus* shows near-isometry in wingspan, negative allometry in humerus, radius and metacarpal IV and positive allometry in WP2–3 relative to body length [16]; (ii) large specimens of *Pterodactylus* have significantly longer necks and skulls, and the metacarpal IV and proximal wing phalanges show positive allometry relative to the antebrachium and to distal wing phalanges [15]; (iii) sinopterine pterosaurs share isometry in the ulna, metacarpal IV and WP1–2, and positive allometry in the femur [29]; and (iv) *Pteranodon* shows negative allometry in tibia relative to femur and positive allometry in metacarpal IV relative to radius/ulna and to WP1 [14].

### (b) Ontogeny of wing planforms

**Figure 3.**
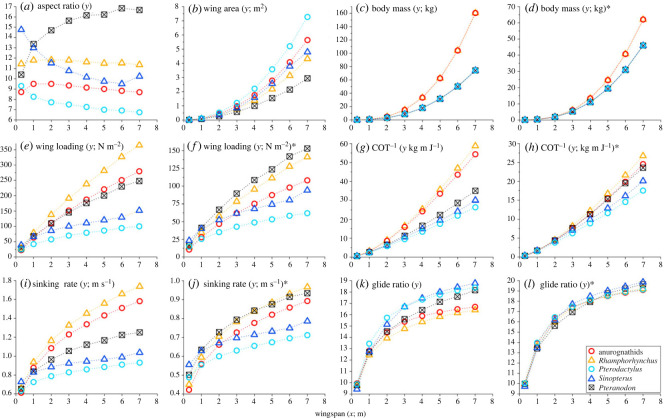
Wing aspect ratio (*a*), wing area (*b*), body mass (*c*,*d*), wing loading (*e*,*f*) and flight performance (*g*–*l*) during growth from an early juvenile of 0.3 m wingspan to a (hypothetical for all pterosaurs except *Pteranodon*) giant adult of 7 m wingspan; modelled using the taxon-specific postures. In (*c*–*l*), models with asterisks are based on the body mass estimation equations by Henderson [34], and those without asterisks are based on the equations by Witton [38]. See electronic supplementary material, figure S3 for corresponding results using the neutral posture.

Based on the recovered allometric growth patterns, wing planforms were reconstructed for each pterosaur during growth from an early juvenile of 0.3 m wingspan to a (hypothetical for non-pterodactyloid pterosaurs, *Pterodactylus* and *Sinopterus*) giant adult of 7 m wingspan. Using taxon-specific postures (see electronic supplementary material, figure S1 for individual wing planforms reconstructed), *Pteranodon* shows the greatest change in wing shape, with aspect ratio increasing from 10.40 to 16.67 (figure 3*a*; electronic supplementary material, table S2). *Sinopterus* also shows striking change in wing shape, but with an overall decrease (from 14.75 to 10.23) in wing aspect ratio. The other smaller-bodied pterosaurs, in contrast, show smaller changes in wing aspect ratio during growth, including relatively consistent values in anurognathids and *Rhamphorhynchus* (with ranges of 8.70–9.51 and 11.36–11.82, respectively) and a decrease (from 9.30 to 6.74) in *Pterodactylus*. Wing areas for all five taxa are similar at small wingspans, but diverge during growth, with *Pteranodon* showing the smallest increase in wing area (figure 3*b*). Wing planform reconstructions using the neutral posture (electronic supplementary material, figure S2) yielded different results, but show similar trends of ontogenetic changes in both wing aspect ratio and area (electronic supplementary material, figure S3*a*,*b* and table S3).

### (c) Ontogeny of flight performance

Based on the scaling equations of wingspan and body mass by Witton [38] and those by Henderson [34], two sets of body mass estimation were generated (figure 3*c*,*d*; electronic supplementary material, tables S4 and S7). Both methods predict that non-pterodactyloid pterosaurs were heavier than pterodactyloids for a given body size and that this difference becomes progressively more pronounced during growth (figure 3*c*,*d*). Equations by Witton [38], however, predict greater values for, and greater differences between, body masses for the two pterosaur groups. As a result, there is little overlap between the two pterosaur groups in wing loading at wingspans >2 m, with non-pterodactyloid pterosaurs being generally more heavy-loaded, irrespective of limb postures (figure 3*e*; electronic supplementary material, figure S3*c*). Estimations based on Henderson [34], however, place the non-pterodactyloid pterosaurs in the wing loading range of the pterodactyloids at wingspans >2 m (using taxon-specific postures; figure 3*f*) or nearly so (using the neutral posture; electronic supplementary material, figure S3*d*). Nevertheless, all models indicate increasing wing loading during growth for all five pterosaurs, caused by (near-)isometry or negative allometry in many wing elements (figure 2) and therefore a slower increase of wing area than the cubic increase of body mass.

Aerodynamic modelling based on taxon-specific postures reveals increasing flight efficiency (COT^−1^) and better gliding performance (based on the increasing glide ratio) during growth for all five pterosaurs (figure 3*g*–*l*; electronic supplementary material, table S4). The somewhat counterintuitive increase in glide ratio despite an increase in sinking rate reflects a faster rate of increase for forward gliding speed than sinking rate, although they both increase with wing loading [39]. As a result, although a gliding adult loses height more rapidly than a juvenile, it glides at a faster speed and therefore can travel a greater distance (i.e. better gliding performance) than the latter. The estimated glide ratios are higher than those for extant tetrapod gliders (glide ratio *ca* 2–4.7) [25]: young pterosaur juveniles are similar to birds of prey (glide ratio *ca* 10–15) [43], but during growth pterosaurs become more comparable to oceanic birds (glide ratio *ca* 13.8–25.1) [44].

These models did not indicate superior flight performance in *Pteranodon* compared with the smaller-bodied pterosaurs in either flight efficiency or gliding performance, irrespective of body mass estimation method. Rather, the models predict a flight efficiency for Pteranodon lower than or comparable to those of non-pterodactyloid pterosaurs (figure 3*g*,*h;* electronic supplementary material, table S5). In particular, for small wingspans (where wing planform is not hypothetical for each of the smaller-bodied pterosaurs), flight efficiencies are similar for all five taxa. In terms of gliding performance, the model based on Witton [38] estimates *Pteranodon* having a glide ratio higher than *Rhamphorhynchus* but lower than *Pterodactylus* at all wingspans, lower than *Sinopterus* at wingspans >2 m (figure 3*k*) and comparable to anurognathids (electronic supplementary material, table S6). By contrast, the model based on Henderson [34] estimates a comparable glide ratio for *Pteranodon* and smaller-bodied pterosaurs (figure 3*l*; electronic supplementary material, table S6).

Aerodynamic modelling using the neutral posture produced consistent results. All aerodynamic indices are associated with similar ontogenetic trends to those based on taxon-specific postures (electronic supplementary material, figure S3). Critically, for both flight efficiency and glide ratio, *Pteranodon* either falls in the range for the smaller taxa or is not significantly different from any of the latter (electronic supplementary material, figure S3 and tables S7–S9).

## 4. Discussion

### (a) No support for linking the giant size of *Pteranodon* with its wing aerodynamics

Based on the allometric growth patterns (figure 2), we reconstructed wing planforms and tested the hypothesis that juvenile *Pteranodon* had an aerodynamically superior wing planform and thus higher flight efficiency and/or better gliding performance that allowed further growth than taxa with smaller adult sizes. Our results do not support this hypothesis. For both flight efficiency and glide ratio, *Pteranodon* either falls in the range for the smaller taxa or is not significantly different from members of the latter at the same wingspans, regardless of body mass estimation method and (taxon-specific versus neutral) limb posture. Further, our aerodynamic models reveal similar ontogenetic changes in all five taxa, i.e. increasing flight efficiency and gliding performance with growth. Increasing flight efficiency with growth is expected, because COT^−1^ correlates with mass for various types of locomotion (including running, flight and swimming), and it is energetically cheaper for a large animal to move a given mass over a particular distance than for a small animal to travel the same distance [45,46]. In terms of gliding performance, all five pterosaurs show increasing glide ratio despite an increasing sinking rate. This indicates that adults lose height more rapidly than juveniles during gliding, but the adults glide at a much faster forward speed and thereby travel further than juveniles.

Body mass estimation for pterosaurs has been notoriously problematic (see [34] for a detailed review) and we show that different body mass estimates can alter various aerodynamic indices (figure 3*e*–*l*; electronic supplementary material, figure S3*c*–*f*). Critically, while both Witton [38] and Henderson [34] predict that non-pterodactyloid pterosaurs were heavier than pterodactyloids of the same sizes, which is sensible given the more extensive pneumatization and tail reduction in the latter group [47,48], the two methods differ considerably in predicting mass differences. This appears to have a broad impact on the estimation of flight performance—a smaller body mass difference (predicted by Henderson [34]) results in less ontogenetic divergence among the five taxa in flight efficiency, sinking rate and glide ratio (figure 3; electronic supplementary material, figure S3). This uncertainty in body mass estimation, however, probably has limited impact on our conclusion that *Pteranodon* had a comparable level of aerodynamic performance to the smaller taxa. This is because (i) our models using either body mass estimation method yielded consistent results, (ii) it is unlikely for the body mass difference between non-pterodactyloid pterosaurs and pterodactyloids to be larger than that predicted by Witton [38] (who predicts that non-pterodactyloid pterosaurs were more than twice as heavy as pterodactyloids at 7 m wingspan), and (iii) if the actual body mass difference between the two pterosaur groups was smaller than predicted by Henderson [34], the ontogenetic divergence in flight performance should be even less between *Pteranodon* and other pterosaurs.

Different limb postures can also alter aerodynamics by changing wing morphology [35,36], as demonstrated by the differences in wing planforms (electronic supplementary material, figures S1 and S2) and aerodynamic estimations (figure 3; electronic supplementary material, figure S3) between the models using taxon-specific and neutral postures. Given that the taxon-specific postures are more accurate/natural as they are aerodynamically more balanced (see Material and methods) than the neutral posture, the consistency between the models suggests that deviation from the natural postures may have a limited impact on our conclusion.

It should be noted that our aerodynamic models were simplified by design to accommodate available fossil data, as in previous studies on pterosaur flight (e.g. [12,25]). We advise caution in interpreting these results because many aspects of pterosaur anatomy remain unclear. These include (but are not limited to) plumage [49,50], muscular wing–body junctions [51], trailing edges of the wing membranes, and the morphology and function of the pro- and uropatagium [37], material properties that relate to shape changes under load [35], and metrics that relate to three-dimensional wing morphology, such as camber of the wing membrane and of the wing bone [52], location of the wing bone relative to the lifting surface [52] and skeletal–muscular configuration of the shoulder girdle [53]. These aspects, however, either are rarely preserved or cannot be determined for fossils and may vary between taxa (e.g. anurognathids had distinct wing fingers capable of flexion at all joints [33]) and even within an individual over its ontogeny (e.g. use of different gaits at different body sizes). These unknowns also prevent further quantification of many other aerodynamic indices than studied here; for instance, quantification of agility and manoeuvrability would require a detailed characterization of inertial properties [54]. In addition, wing planform and flight performance could be highly dynamic, as modern birds use in-flight wing morphing for transition between stable and unstable states and thereby dynamically trade between flight efficiency and manoeuvrability [54]. This would certainly have been possible in pterosaurs given the muscle fascia layer in the wing membranes and the ability to move both the fore- and hindlimbs at various joints [1,55].

Nevertheless, we argue that the available evidence suggests that *Pteranodon* had comparable level of aerodynamic performance to the small taxa at similar wingspans. The dramatic increase in wing loading with growth, observed here in pterosaurs representing diverse lineages and wing planforms, was likely a universal theme for pterosaur ontogeny. Indeed, similar wing shapes between juveniles and adults have been reported in many pterosaurs (e.g. [7,8,16,17,25]), suggesting (near-)isometric wing growth that is much slower than the cubic growth of body mass. Consequently, juveniles would have had low wing loadings that promoted slow, manoeuvrable flapping flight, whereas the heavier adults would have had improved gliding performance (via higher glide speed and stability in high wind conditions) and higher flight efficiency [11,39,45,46]. This confirms previous inferences of different flight behaviours in juveniles and adults of *Rhamphorhynchus* [16]; similar flight performance changes during growth have also been inferred for the pterodactyloids *Sinopterus* and *Pterodaustro* [25].

### (b) Precocial–altricial growth strategy and its link to pterosaur body size

*Pteranodon* shows a unique growth pattern that hints at a different developmental strategy from that of the smaller-bodied pterosaurs. Among the smaller-bodied pterosaurs, all four taxa show negative allometry or isometry in the proximal elements of forelimbs and three taxa show this feature in hindlimbs, whereas positive allometry was detected in the proximal elements of both fore- and hindlimbs of *Pteranodon* (figure 2). This indicates differential growth rates in these limb elements and possibly the associated major muscle groups, as observed in developing birds [23,24]. In modern endotherms (birds and mammals), there is a physiological trade-off between growth rate and functional maturity of tissues owing to their overall high growth rate [21,56,57]. This leads to the precocial–altricial developmental spectrum, that is, the high–low degree of functional maturity at hatching (or birth) coupled with low–high postnatal growth rates and negative/isometric–positive growth allometry in locomotors [21–23].

Similar trade-offs between growth rate and functional maturity may have also regulated pterosaur ontogeny, since several independent lines of evidence indicate that pterosaurs had high growth rates that were comparable to those of known endotherms [58–60]. The observed divergent growth trajectories in the proximal elements of both forelimbs and hindlimbs, which are critical elements for powering both flight and terrestrial locomotion, may thus reflect precocial development in the smaller-bodied pterosaurs and more altricial development in *Pteranodon*. Indeed, in line with the interpreted precociality, early juveniles of anurognathids and hatchlings of *Sinopterus* had considerably high bone strength for the humerus [25,61]; ontogenetic changes in the food-gathering apparatus of *Rhamphorhynchus* and *Pterodactylus*, including changing tooth morphology in the former and positive allometric growth in the skull and neck length in both taxa (electronic supplementary material, table S2), suggest that precocial juveniles may have fed on prey different from that of adults [1,15].

The interpreted altricial development of *Pteranodon* is consistent with our finding that its wing planform is not aerodynamically superior in growth to the smaller-bodied pterosaurs. If the reverse is true, that is, early juveniles of *Pteranodon* had an aerodynamically superior wing planform, it would be reasonable to expect their wings to be as functional, with a similar (if not higher) degree of precociality, as in the smaller-bodied pterosaurs; instead, *Pteranodon* is more altricial. The detected altricial signal for *Pteranodon,* however, does not necessarily preclude its flight capability relatively early in growth. Indeed, the elongated wing morphology is evidently present in the smallest specimen, with an estimated wingspan of 1.76 m [31], and according to our results (figure 2; electronic supplementary material, figure S1), may have also been present in smaller individuals, possibly a remnant heritage from its precocial ancestors. This contrasts with altricial birds and bats, which attain their large, functional wings only at, or close to, adult sizes [23,62,63]. Further, given the low wing loading of early juveniles, their bone density would need to be considerably lower to reduce wing bone strength to levels impossible for flight [25,64]. Early juveniles of *Pteranodon* therefore may have been facultative flyers that employed only occasional flights to maintain a low tissue maturity and a high growth rate, as seen in some juvenile birds today [65].

It should be noted that the interpreted altricial development of *Pteranodon* is based on the assumption that the recovered positive allometric growth in *Pteranodon* can be extrapolated to smaller juveniles (less than 1.76 m wingspan) for which no fossils are currently available. The alternative to this assumption is that small juveniles of *Pteranodon* grew differently from their older counterparts; they might have been precocial like the smaller taxa, having well-developed (and functional) limbs at hatching with isometric/negative allometric growth in early ontogenetic stages and then shifted to the observed positive allometric growth at later stages. This, however, would have required a physiological switch from high to low tissue maturity in the proximal elements of both forelimbs and hindlimbs, which is currently unknown in modern animals [57], and it is unclear whether it is physiologically possible. We therefore consider it more likely that the detected altricial signal can be extrapolated to early ontogenetic stages of *Pteranodon*, although growth rates (and coefficients of growth allometry) may have been heterochronic, possibly with the fastest growth occurring in early ontogenetic stages.

(Super-)precocial flight has been suggested for many pterosaurs of diverse clades [1,7,8,25] and may represent the plesiomorphic condition in pterosaurs. This condition in turn may have posed constraining effects on adult sizes of early pterosaurs. Precocial locomotion has often evolved in response to high predation rates on relatively vulnerable juveniles [66–68]; for pterosaurs, the juveniles may have also suffered from high rates of flight accidents [69]. The high risk of mortality in juveniles might have favoured selection for a shortened period of this vulnerable stage and early termination of growth and thereby constrained adult sizes [21,70]. Also, given the physiological trade-off between growth rate and maturity, precocial development could have induced ontogenetic canalization, that is, retention of juvenile phenotypes into adulthood [23,71], including a relatively small body size.

By contrast, post-hatching parental care in altricial species would reduce the risk of mortality in juveniles [72]; it would also relieve the mechanical demand in juveniles by providing protection and/or food, and thereby facilitate bypassing the potential effects of ontogenetic canalization [23]. Although evidence for specific modes of parental care in pterosaurs is lacking, the detection of an altricial signal in the development of the limb elements that were critical for powering both flight and terrestrial locomotion implies an enhanced level of parental care in *Pteranodon* compared with the smaller-bodied pterosaurs. This may have been the key innovation by *Pteranodon*, and by extension, perhaps other giant pterosaurs, to break the body size constraints on their precocial counterparts. By allowing further morphological departure from the juvenile condition [23], altriciality might have enabled development of the derived flight anatomies seen in giant pterosaurs, such as enlarged developed notaria, enlarged deltopectoral crest, robust joints and more extensive pneumatization and lightening of the skeleton [10,47,48,73].

Our proposed altricial developmental strategy for giant pterosaurs is an intrinsic factor that can explain growth to larger sizes. The extrinsic factor, i.e. the availability of ecological niches suitable for large-bodied pterosaurs, is also critical. All giant pterosaurs probably preferred soaring conditions where atmospheric movements can be exploited [10]. For instance, it is widely accepted that *Pteranodon* was a dynamic soarer living in open marine settings, gliding long distances through exploitation of updrafts and wind currents [10,38], whereas *Quetzalcoatlus* was likely a static soarer adapted for thermal soaring in terrestrial environments [10]. Giant pterosaurs would have also preferred open environments (e.g. coastal, pelagic environments or inland but without dense forests), given their large sizes (even on the ground with wings folded), thin-walled wing bones vulnerable to impacts [1,5,74] and overall high flight speed and low manoeuvrability due to their high wing loading [11,39]. Critically, there seems to be a general lack of competition with and/or predation on the giant pterosaurs in these environments [1–3,75]; this vacancy would have set the stage for the emergence of giant pterosaurs, expediting their invasion to large-bodied niches.

## 5. Conclusion

Our research shows that during growth, *Pteranodon* was aerodynamically comparable to smaller-bodied pterosaurs with the same wingspans, thus rejecting the hypothesis that an aerodynamically superior wing planform enabled *Pteranodon* growth to its giant size. *Pteranodon* did, however, show a unique allometric growth pattern that hints at a different developmental strategy. Unlike the smaller-bodied pterosaurs, *Pteranodon* had positive allometric growth in the proximal elements of both the fore- and hindlimbs and possibly the associated major muscle groups, all of which were critical for powering flight and terrestrial locomotion. This indicates that *Pteranodon* was more altricial than the smaller-bodied pterosaurs, which likely had (super-)precocial flight, as has been interpreted for many other non-giant pterosaurs of diverse clades [1,7,8,25]. Therefore, a shift from a plesiomorphic precocial state towards a derived state of enhanced parental care may have relaxed the constraints of small body sizes [21,23]. This, coupled with vacant niches for large-bodied animals [1–3,75], may have shaped the evolution of giant pterosaurs.

## Data Availability

The data are provided in the electronic supplementary material [76].
